# Managing relational autonomy in interactions: People with intellectual disabilities

**DOI:** 10.1111/jar.12595

**Published:** 2019-04-12

**Authors:** Sandra Dowling, Val Williams, Joe Webb, Marina Gall, Deborah Worrall

**Affiliations:** ^1^ Norah Fry Centre for Disability Studies, School for Policy Studies University of Bristol Bristol UK; ^2^ Centre for Academic Primary Care University of Bristol Bristol UK; ^3^ School of Education University of Bristol Bristol UK

**Keywords:** conversation analysis, decision making, interaction, mental capacity, relational autonomy, support practices

## Abstract

**Background:**

This article is about interactions that occur when someone with intellectual disabilities is engaged in everyday activities with a personal assistant (PA) or a support worker.

**Method:**

We examine the detail of nine hours of naturally occurring video‐recorded interactions, to explore how “relational autonomy” is done in practice. Nine people with ID and seven staff took part in the research, which took place in England from 2016–17.

**Results:**

We selected six extracts to illustrate different types of joint decision‐making. Informed by inclusive research with a drama group of people with intellectual disabilities, we focus on the ways in which (a) future plans are discussed; (b) choices are offered during an activity; (c) people reflect on their decisions.

**Conclusion:**

The article concludes with discussion about the teaching and learning content of choice‐making, on relational autonomy, and the practice learning for PAs, support workers and for people with intellectual disabilities.

## 1 INTRODUCTION

In this article, we explore interactional practices which involve a person with intellectual disability and their conversation partner. In England, people with intellectual disabilities sometimes have personal assistants (PAs) when they are paid for by the individual's personal budget. However, since the focus here is on social interaction, a “conversation partner” could be anyone, maybe a volunteer, a friend or a formal paid support worker. At an international level, not all countries have a welfare system designed to support disabled people in their independent living (Priestley, 2001, p. 3). What is truly global is communication itself. We are interested here in how these interactions support or undermine the autonomy and control of the individual, and how they are played out on a day‐to‐day basis in real time.

Pervasive notions of autonomy in Western culture emphasize the desirability of independence and self‐determination. Self‐reliance is viewed as an individual achievement and one that many disabled people, who may require assistance with daily living, are not able to aspire to. However, “choice and control” are salient themes in policy about personalized services for all disabled people in the UK (Glendinning, 2008) and have particularly been highlighted in intellectual disability services, where people's lives have traditionally been dominated by protection (Stainton & Boyce, 2004). We know that these are also seen as important issues in many other countries, including Malta (Callus & Bonelli, 2017), Australia (Bigby, Whiteside, & Douglas, 2017; Dowse, 2009) and the United States (Schelly, 2008). Many of these authors turn to the UN Convention on the Rights of Persons with Disabilities (UN, 2007) which enshrines the right to legal capacity for disabled people in Article 12, and further the right to have all necessary supports to exercise that capacity. This has been the basis for legal frameworks such as the 2005 Mental Capacity Act in England and Wales, and for models of supported decision making (SDM) (Bach, 2017; Bigby et al., 2017), a process “by which a third party …assists or helps an individual with an intellectual or cognitive disability to make legally enforceable decisions by themselves” (Devi, 2013, pp 792–793). It should not be assumed, simply because someone has an intellectual disability, that they cannot make a decision. Conversely, well‐intentioned support workers may hold back from offering support to a person with an intellectual disability in managing decisions in order to ensure they are able to exercise their autonomy freely and without coercion. In this article, we are concerned with moments in which the principles of autonomy are translated into interaction with support workers, during the course of everyday life. As Beadle‐Brown (2015) suggests, we do not need new legislation, as much as the impetus to change the “culture of support” (p. 26).

“Relational autonomy” highlights how the ability to have control and agency in one's life requires interdependence, not isolated independence (Morris, 2004; Perkins, Ball, Whittington, & Hollingsworth, 2012). It is regularly highlighted in research relating to people with intellectual disabilities (Johnson, Walmsley, & Wolfe, 2010), and builds on ideas in disability theory, particularly the “relational” model of disability (Callus & Bonelli, 2017; Goodley, 2011; Tøssebro, 2013). If disability is situated at the interface of impairment and the environment with which a person comes into contact, then it makes sense to look towards relationships (Forrester‐Jones et al., 2006; Jamieson, Theodore, & Raczka, 2016; Simplican, Leader, Kosciulek, & Leahy, 2015; Williams & Porter, 2017) as an important place in which disabling or enabling effects will play out. Most research in this area is based on interviews or focus groups (Bigby et al., 2017; Jamieson et al., 2016), where participants are asked to talk about the ways in which they make joint decisions in the context of “a good working relationship,” although Dunn, Clare, and Holland (2010) did include some ethnographic observation when asking support workers about how they made decisions *for *people with intellectual disabilities. Authors more frequently discuss relational autonomy as an abstract concept, rather than considering in detail the variety of ways in which it plays out in interaction, which is the path followed in this article.

There is a small collection of detailed research in Conversation Analysis exploring the “institutional” inequalities which can be observed in interactions involving people with intellectual disabilities. Antaki, Walton, and Finlay (2007) have shown how conversations with people with intellectual disabilities can construct them as incompetent. They have also analysed instructions given by support workers (Antaki, 2013; Antaki & Kent, 2012) and the ways in which choices may be undermined in everyday conversations in the home (Antaki, Finlay, Walton, & Pate, 2008). Williams (2011) highlighted similar findings in one‐to‐one interactions, drawing on data collected in 2002–2004. The aim there was to capture more empowering and positive interactions with personal assistants. Similarly, Jepson (2011) examined the detail of conversations between support workers and people with intellectual disabilities specifically in the light of the Mental Capacity Act in England, finding evidence that the third principle (that “unwise decisions” do not equate with lack of capacity) was easily flouted, with supporters advising, persuading and guiding choices. For instance, healthy eating principles could take precedence over someone's initial choice.

The current paper thus builds on this interest in the actual interactions which take place when a person with intellectual disability is receiving support. Unlike Jepson (2011) and Antaki et al. (2008), we turn to contexts where there is a presumption that the person with intellectual disability will be “empowered” by employing their own PA, or by being in control of their own social enterprise. Our questions in this paper have a central focus on different strategies for doing “relational autonomy” which emerge from interactions. Our research questions are as follows:

- In what ways do communication partners undermine or support decision making by people with intellectual disabilities?
- How do those decisions play out in the course of everyday activities and in the context of a relationship between the two people?
- What implications do these findings have for personal assistants (PAs) and others who support people with intellectual disabilities?

## 2 METHODOLOGY

The data in this article were collected during a three‐year (2015–2018) research programme "Getting Things Changed" which was about understanding and changing disabling social practices (Williams et al., 2018). The various strands of our project conceptualize social practices and change in different ways, and the present paper is based on a conversation analytic approach (CA) (Sidnell & Stivers, 2012). The main features of the CA approach are that data are collected which are “naturally occurring,” with an interest in how the fine detail of communication is organized. It should be noted however that our goal here is to present our analysis to a wider audience, and so the transcription conventions familiar in CA have not been used. This paper aims instead to use CA insights to examine a specific, institutional context for people with intellectual disabilities, and we have made an attempt at an accessible account for readers without specific CA knowledge.

Conversation analysis is epistemologically distinct from many qualitative methodologies, which are about people's intentions, feelings and reflections. CA by contrast is more interested in the structure and shape of talk, and the actions performed in everyday interactions (Schegloff, 2007). Those actions (such as questions‐answers; corrections or praise‐giving) play out in certain revealing ways when a person with intellectual disability is talking with a conversation partner. In the current project, 9 hr, 6 min of video data were collected with nine people with intellectual disabilities in England, in different contexts, by each of the first three authors. The primary interactional context is a one‐to‐one conversation with a personal assistant, with a secondary setting being a workshop setting where two members of staff were supporting people with intellectual disabilities to carry out craft work.

The participants with intellectual disability all had expressive language, and most would be described in the UK as having moderate learning disabilities. All names used in this paper are anonymized, and details of participants are given in Table 1.

**Table 1:** Participants

Pseudonym	Age bracket	Everyday life and independence	Communicating choices	Length of relationship with communication partner(s)
Paul	18–24	Lives in parents’ home with PAs after college in evening	Can express own choices and preferences	Rik (4 months) and Ann (about 1 year)
Katie	18–24	Lives in parents’ home, goes to college ‐ but filmed in PA's home	Can express own choices, can plan ahead for herself	Lola: 3–4 years
Lyn	41–49	Filmed in craft workshop: a beginner in some skills there	Quiet, takes time to respond, but good understanding	Sally: 1 year
Wendy	41–49	Lives in own flat in sheltered housing block	Sophisticated communicator, can express what she wants PAs to do	Sarah: 3 months
Jen	25–30	Lives in own flat, with PA visits	Sophisticated communicator, can plan and manage own life	Rachel: 5–6 months
Anna	31–35	Lives in own flat with a few hours PA support	Vocal self‐advocate: can choose and plan her own life	Pamela: 9 months
Jon	36–40	Filmed in craft workshop: skilled potter	Vocal self‐advocate: can choose and plan within the workshop	Sally: 1 year
Fay	25–30	Filmed in craft workshop: skilled designer	Can express own choices and preferences	Leanne and Sally: 1 year
Marion	41–49	Filmed in craft workshop: skilled designer	Quiet, but chooses own activities	Sally: 1 year

Making videos with people with intellectual disabilities has many ethical implications, and beyond the usual informed consent, we were careful to develop research protocols in which participants could familiarize themselves and find out more about the research before agreeing to take part, using accessible information and a recruitment video on some occasions. We conducted initial interviews with those who agreed, asking both the PA and the person with intellectual disability what mattered most to them when they interacted, and we also took videos back to those we had filmed, in order to ask them about their feelings and reactions to the video. Participants were given the option of deleting specific parts of their data, but in fact, no‐one chose to do so. This strand of our research was approved by the Social Care Research Ethics Committee for England.

Video data collection was carried out following lengthy periods of familiarization with the researcher. The people with intellectual disability chose when to switch on the small hand‐held camcorder, and what activities could be recorded, with the researcher where possible withdrawing from the scene. The video data included conversations at home during domestic activities, planning and carrying out food shopping, going for a walk, playing outdoors sport, preparing food, eating in a café, disassembling furniture to return it to a store, caring for a baby, and conversations in the craft workshop.

Extracts for analysis were selected after repeated watching of the raw data, and discussions within the team. Unusually for Conversation Analysis, however, we wished to ensure that the points we noticed in the data were relevant and important for people with intellectual disabilities themselves; during this stage, therefore, we bought in the services of a company of disabled actors, the Misfits Theatre Company (2018), who had previously been engaged as project advisors. They helped us by reconstructing and role‐playing scenes selected as potentially interesting by the authors. Six meetings were organized and filmed by Sandra Dowling with 6–8 actors, all of whom had experience in different ways of interacting with supporters or personal assistants. Their insights informed the analysis in this paper, and in the final stages of our project, they produced a video for other people with intellectual disabilities who employ PAs which includes some of their dramatic re‐constructions. We mention some of their specific points throughout the paper.

During this stage, short extracts were isolated and transcribed in detail, in order to focus specifically on patterns of turn‐taking and sequencing. In CA what matters is not just “what” someone says, but how each utterance is linked to and reveals how the previous utterance was taken (Sidnell & Stivers, 2012). We took as wide a view as possible of what might constitute relational autonomy, not starting with any fixed ideas, and made a collection of 84 instances where the autonomy, choice or decision of the person with intellectual disability was made “visible” in the talk or the embodied action. We then sorted them into a loose classification system, first of all according to who initiated the sequence, and secondly according to what seemed to be the main outcome achieved. The current article builds on this wider collection in order to focus back down on specific extracts which have been chosen to develop insights into the various ways in which relational autonomy is manifested in action.

## 3 FINDINGS

### 3.1 Planning for future activities

When a person with intellectual disability is with their PA or supporter, their possible plans of action are frequently negotiated. Our first video extract takes place in a park, where we see three people walking along a path, one of them trailing behind the others. This is Paul who is accompanied by two personal assistants, Rik and Ann.

**Table:** 

**Extract 1**		
01	Paul	And this called a tennis court
02		(*gestures towards tennis court)*
03	Rik	Yes, absolutely. We'll come here and
04		do that next time, yeah?
05	Paul	Yeah

One might assume from Line 1 that Paul is simply observing something and making conversation. However, that is not how his remark is taken. Although Rik affirms the point that it is in fact a tennis court, he immediately goes on to suggest a plan of action for “next time” they are in the park. Planning forward, noticing what Paul might want, and literally putting that next activity on the agenda—all these things appear to be routine in the conversation that Paul has with his PAs on his walk. Sometimes the suggestions come from Paul himself, sometimes they are jointly constructed as in Extract 1, and at other times, interactional trouble ensues, as in the following extract.

The three people are now a bit further on in the park, with Paul walking behind his PAs, and he cuts into a conversation between his two PAs about the route they are taking via the park, to introduce the topic of dancing.

**Table:** 

**Extract 2**		
01	Paul	We will talked about the way err (.)
02		dancing around.
03	Ann	You want to talk about dancing
04		around? *(walking in*
05		*front of Paul)*
06	Paul	Yeah *(another pause)*
07	Ann	What about your‐ y‐you could tell
08		Lisa (*camera person)*
09		about umm your dancing (*glances*
10		*back towards Paul*).
11		You do on a Monday
12	Paul	Lisa? (*speaking quickly) *W‐w where
13		we're going to do it do it on next
14		Sunday *(catches up*
15		*with Ann and Rik at this point)*
16	Ann	On a Sunday? We do it on a
17		Monday we do tha‐ or are
18		you going dancing on Sunday?
19		(*Paul drops behind)*.
20	Paul	I'm going DOWN in the thunder
21		(*gestures downwards*
22		*with one hand)*
23	Ann	You're going dancing in the
24		thunder? (*reaches into her*
25		*bag for phone)*
26	Paul	No I'm going DOWN in the
27		thunder (*hand gesture downwards)*
28	Ann	You're going down in the thunder?
29	Paul	Yeah
30	Ann	Down where?
31	Paul	Down in the (s)under I told you.

Paul clearly orients to talking about his future plans when he is with his PAs, and that might well be because they are ordinarily entailed in organizing these activities for him. In Line 3 Ann quickly picks up on the “dancing” and then at Line 7, suggests that Paul could tell the camera person about his dancing on Mondays. However, he corrects her assertion that dancing takes place on a Monday, saying quite clearly that it might happen on Sunday, a day when his PAs would not be working with him. The dancing plan is something that *he *may know about, outside his PAs’ domain of knowledge, and by mentioning it, he creates himself as an independent decision maker with the epistemic right to know about his own life experience. People normatively treat themselves, and are treated by others, as having primary rights to know and describe things in their own life and experience (Heritage, 2012; Sacks, 1984). A point to bear in mind then is that Paul's individual autonomy is only visible when he counters something his PAs suggest.

Visually, we also noticed in this interaction that both PAs are walking slightly in front of Paul, although Ann turns towards him as he starts up the conversation. In a subsequent interview, Ann explained that they always walk in front of Paul to encourage him to keep moving. However, body position is significant for interaction, and CA research has shown how the coordination of body movements is important for “the jointness of a decision” (Stevanovic et al., 2017, p. 36). When walking and talking, moreover, actions involving objects or place may compete with verbal interaction; at line 24 Ann was pulling her smartphone out of her shoulder bag, as she turned to acknowledge Paul's comment. “Rik” the second PA was on the far side of Ann, and was looking away, and at this point Paul's talk about “Sunday” is hearable as “Sunder” or even as “Thunder.” As the weather was uncertain, the “thunder” interpretation is checked out by Ann first at line 23, who then embarks on a confused interaction to determine whether Paul is talking about the here‐and‐now (the weather) or about his future plan for dancing. At the same time, we can equally see how this is starting to cast Paul as an incompetent or muddled decision maker (Robinson, 2006), although his frustrated “I told you” at 31 implies that Ann is the muddled party.

When this extract was shown to the Misfits Theatre Company (2018) members they strongly felt that Paul's frustration was grounded in the fact that he was being ignored both physically and in terms of his own meaning. In fact, following Extract 2, he playfully disappeared from sight, forcing his PAs to play a game of hide‐and‐seek with him. A resulting problem was that what Paul did to get attention could be considered difficult or “naughty,” and that ultimately would not serve to equalize the relationship that he had with his PAs, but would rather create or reproduce a narrative supporting his dependence and need for surveillance.

### 3.2 The foregrounding of choice

Unlike Paul's conversations, which are about the negotiation of *future* action, choice can be “offered” during the course of ongoing activity, and is often closely tied to the accomplishment of the activity. In mundane conversation, we all offer choices to others. However, if someone has made a choice, and we repeat the original offer with something like “Which one do you want?” the natural implication is that the person's first choice was wrong. In Antaki et al.'s (2008) example, the person with intellectual disability goes back to make the opposite choice to his original one, and we have a very similar example where a young woman called “Katie” is in the kitchen making a salad with her PA, “Lola.” Katie is given the choice of two knives and immediately leans forward to touch one of them. However, Lola explicitly instructs her about how to make a better choice:

**Table:** 

**Extract 3**		
01	Lola	that you have to look not
02		just go eeny meeny meeny
03		(*walking back to work surface,*
04		*holds knives up again)*
05		which one do you want
06		gonna go with the best
07	Katie	*points to smaller knife*.
08		That one *(PA immediately*
09		*takes other knife back*)
10		little one (.) little
11	Lola	right

Lola's explanation at Line 1 of how, in general, choices should be made, implies strongly that Katie's choice was a random one (“eeny‐meeny” being a way of choosing something by chance). What happens as a result? At line 8, Katie reacts to the message that her first choice was in some respect wrong. Not only should she put more thought into the choice, but she probably ought to choose the other knife! She articulates here the reason for that choice, that the correct knife to choose was a “little” one, and Lola accepts this with a “right” as she replaces the knife, very much as a teacher would (Hellermann, 2005).

In other examples, a choice action by a person with intellectual disability is immediately followed by negative feedback. For instance, Paul (the young man walking in the park) wanted to go on a motorbike at one point, but was told “No, that doesn't belong to us.” A young woman choosing meat for a curry wanted to select “mince” but was told there was not much mince there, and that she would be “better off with chunks of meat,” and a young woman in the workshop who had selected items for an upcoming display, was told that “we want the right colours.” All the people in these examples had actively chosen to place themselves in learning situations, and so the feedback they receive is perfectly reasonable, even though it places them in a K‐ position. On such occasions, there is a demonstrable shared understanding of what is right or appropriate, and that in itself can consitute a type of relational autonomy.

In their interviews, our participants often discussed the extent to which the PA role included teaching or instructing them to manage everyday activities. We can see in Extract 3 how Lola seized on the moment, during an ongoing joint activity, to teach Katie about the use of sharp knives. In CA, this might be referred to as a “teachable” (Cohen, Clark, Lawson, Casucci, & Flocke, 2011; Slembrouck & Hall, 2017). The third principle of the MCA, about “unwise decisions” not being equated with a lack of capacity, often seems to be flouted in favour of using a decision moment as a chance to practise “getting it right” (see also Jepson, 2011). The Misfits Theatre Group members included a role‐play in their training video which was about someone being denied the choice of a biscuit by her PA. They discussed at length whether such choices should be left to the person with intellectual disability, and concluded that there were occasions on which the PA needed to step in.

Relational autonomy takes place, in other words, in a wider context of roles, situations and identities—all relevant to and visible in the interaction. For instance, some of our data was filmed in a work environment, where the learning of appropriate skills was part of the overt aim. Sally here is the support worker, who is preparing clay with Jon, one of the clients, while Lyn is waiting at the pottery wheel.

**Table:** 

**Extract 4**		
01	Sal	how about you Lynn what do you
02		reckon you'd wanna make?
03		(*Lyn looks at Sal, pause)*
04		It's got to be round.
05	Lyn	(*unclear)*
06	Sal	yeah
07	Lyn	I saw it on a DVD
08	Sal	a bowl, a plate – or – a vase, but it
09		wouldn't be a very good
10		vase. (*Long pause). *A dish?
11	Jon	Saucer?
12	Lyn	A mug?
13	Sal	You want a mug?
14	Lyn	Yes I've had enough cups
15	Sal	Yeah? A cup's just a mug without a
16		handle isn't it? (*long pause)*
17		OK we'll have a go and look at the
18		shapes and see if inspiration
19		strikes you.

The first offer Sally makes to Lyn is a very open one, at line 1, and it is notable how she pauses each time to allow Lyn to think before she responds. This occurs without trouble, since Sally is glancing back at the clay in her hands while she addresses Lyn. However, at Line 4, she follows up the open offer with the restriction that the choice has got to be of something “round,” before Lyn has actually made a verbal response. This is unlike Extract 3, where the correction by the support worker came after the immediate response by Katie about the knife. Thus, instead of Lyn appearing incompetent, she has already been given the prop she may need to make a good decision here. Indeed, it could be said that Lyn has already learnt the principle which Katie was being taught, that it is good to stop and think before responding with an immediate choice. It is only when Lyn then hesitates and is unclear about her response in lines 5 and 7, that Sally uses her second strategy of naming some alternative possibilities at line 8: “a bowl, a plate, a vase.” Jon then helps with this by suggesting a “saucer,” and this leads to the exchange about a mug or a cup. Again, it is interesting how Sally both endorses Lyn's choice, but does not commit her to the choice in any way. Instead, she defers the decision to a later moment, when “inspiration strikes you,” implying that there may be many correct and possible objects which Lyn might choose.

Teachable moments occur frequently during these interactions, and of course there are different implications about getting it “right” or “wrong.” Maybe it did not matter too much what object or shape Lyn chose for her pot, whereas the choice of a knife was part of what Katie had to learn in order to be safe in the kitchen. The practical point to take from this though is that it is often useful for the supporter to (a) spell out what the choice is, and the restrictions associated with that choice, (b) when appropriate, endorse a deferral of the decision, and (c) “feed” a decision with alternatives, both verbal and visual. It is the sequential position of each of these strategies which matters, each utterance by the support worker revealing how she has listened to and is responding to the person with intellectual disability.

### 3.3 Making and discussing decisions during activity

Thus far, relational autonomy has been displayed in activity contexts: the decisions being made are closely tied to the ongoing action, or as in Paul's case, are talked about in the context of an ongoing action. We have written elsewhere (Antaki & Webb, in press) about the possible tension between completing the activity and the conversation itself. There were some particularly interesting examples in the data of more “reflective” talk about a decision which may have already been made, and this type of talk could be initiated either by the person with intellectual disability or the PA. In Extract 5, Wendy had already asked her PA, Sarah, to disassemble and pack some furniture she had previously bought, so that it could be returned to the store.

**Table:** 

**Extract 5**		
01	Sar	No we'll send them back and see what
02		happens alright
03		Wendy? We'll see what happens, alright?
04	Wen	Yeah, and then‐ and then w‐don't be
05		surprised if they say no
06	Sar	Right we'll go from there and w‐
07	Wen	Is that what you mean?
08	Sar	Yeah we'll just go from there and we'll just
09		see what they say.
10	Wen	But don't worry it's my my f‐ my responsibil‐
11		you know don't
12		worry, if I lose the money it's only thirty quid.
13	Sar	So, let's just send them back and see what
14		happens.
15		Alright? At the end of the day, they're split
16		and they shouldn't have …
17	Wen	It was split but I wish I'd checked
18	Sar	…been like that. I didn't even notice see?

Throughout this conversation, Sarah is sitting on the floor with tools to take the furniture apart, while Wendy is picking up plastic wrappers and putting them down beside Sarah. An important point this illustrates is that autonomy is not necessarily about “doing the job” which you have decided on. The relational element of this decision is achieved precisely by Sarah actually carrying out the mechanics of the task, watched and commented on by Wendy. There is a jointness both in the activity and in ownership of the decision, with Sarah's use of “we” from Line 1 onwards, despite Wendy's overt reassurance at line 10: “don't worry it's my my f‐ my responsibil(ity).” Although the decision has been “jointly” made, it is in fact Wendy who stands to lose thirty “quid” (pounds), which she brushes aside as unimportant. Once that decision has been made to send them back and “see what happens” (Line 13), there is further discussion of how they had arrived at this point, with Sarah pointing out that the furniture was faulty, and Wendy taking on the moral responsibility of checking (which she admits she had not done). Even that, though, is seen as a joint accomplishment, with Sarah too admitting that she had not noticed the fault.

Throughout their interactions, this pair engaged in commentaries both on the ongoing action, but also on the reasons for it, and the steps taken in making decisions along the way. A close relationship such as theirs is clearly not only the responsibility of the PA; instead, one can see how decisions are finessed and mulled over, at the instigation of the person with intellectual disability.

### 3.4 Reflecting on factors in a decision

If a person with intellectual disability needs support to exercise their autonomy, one might expect them to be learning from interactions about choice, so that their skills to make “wise decisions” would improve. To some extent that seemed to be happening in Extract 5, where Wendy was learning from her mistake that she should have “checked” the furniture she had bought. We were interested to examine other occasions on which the person with intellectual disability started to talk through the contingencies and background, regarding both their past and their projected future decisions. Extract 6, like much of our data, took place in a kitchen, but related to situations outside the here‐and‐now, in this case the forthcoming visit of “Den” the boiler repair man.

**Table:** 

**Extract 6**		
01	Jen	me dad might be coming as well a
02		bit later on because
03	Rac	oh I'm not letting him in
04	Jen	heh heh Just because y'know cos
05		if Den if Den comes about two,
06		I don't know how long it's going
07		to take to set up but because
08		you know I don't understand
09		things, I don't want him to like
10		take advantage or anything
11	Rac	*looks at Jen and nods*
12	Jen	so probably best if I have dad
13		around or something.
14	Rac	Yeah

Jen's account of why she wants her dad in the house (lines 4–10) is interesting, since it is not immediately clear what that does in the interaction with her PA Rachel. In fact, Rachel's first reaction to Jen's mention of her dad is to make a joke (“I'm not letting him in”) which is picked up with laughter by Jen in line 4. Of course, the “letting dad in” implies that Rachel will still be around at two o'clock, and it could be that which prompts Jen's subsequent justification of her need to have not only Rachel, but also her dad in the house at two o'clock, when a friend “Den” is coming over to mend her boiler. She relates this explicitly to her own need to have help in understanding things, and Rachel affirms that with a nod, and a “yeah.” This small interchange thus serves simultaneously the purpose of planning for Rachel's support and help, while also displaying Jen's own awareness of how to make a wise decision relating to her needs.

What is evident in both Extracts 5 and 6 is that the person with intellectual disability can be heard re‐shaping their own idea, checking it out, receiving ideas about the reasoning behind the decision—or in fact articulating it themselves. There is a real sense in these instances that the PA is supporting the capability of the person with intellectual disability to make a wise decision, by giving advice, implying a sequence of reasoning via the questions asked, or simply by listening to and affirming a decision which has been made.

## 4 REFLECTIONS AND DISCUSSION

It is a truism to state that people with intellectual disabilities need trusted relationships to make good decisions, (Williams & Porter,^2017^) and we only start to learn more about the shape of “relational autonomy” by observing it in practice during everyday life. One striking finding in this paper was that communication partners tended to seek out what we have called “teachable moments,” where attention is directed more towards improving the capacity of a person with intellectual disability, than in determining what they really want. We frequently heard a conversation partner asking a question to which they already knew the answer. This is perhaps most manifestly evident in, and an enduring feature of, teacher–student interactions (Hellermann, 2005). In these instances, as in Extract 3 in this paper, this practice casts the two speakers in pedagogical roles of “teacher” and “learner,” and makes explicit their lack of equality regarding specific territories of knowledge.

Supporters and PAs were routinely offering, but then rejecting or correcting, choices made by the person with intellectual disability, or implying that the initial response was wrong, as in Antaki et al. (2008). One conclusion would be that these supporters should reflect upon the third principle of the MCA, which indicates that making an unwise decision does not equate with a lack of capacity. Simply offering someone a choice implies or reinforces the fact the individual with intellectual disability could be perceived to have less epistemic authority (Heritage, 2012), and therefore creates/recreates dependence in those there to support them. Nevertheless, our analysis revealed some important practical implications about how these teachable moments could proceed successfully, without undermining the autonomy of the person with intellectual disability. To summarize:

- Activities give natural opportunities for “teachable” moments, where choice can be offered, especially where one participant knows more about the activity than the other.
- Giving immediate negative feedback is sometimes appropriate in that moment, but can result in undermining the confidence of the person with intellectual disability to learn from their own experience in future.
- Foregrounding any restriction or constraint on the decision is often appropriate and may help the person with intellectual disability to make a good decision.
- Decisions do not always have to be made here‐and‐now, but could be deferred to give a longer time to consider options.

Nearly all the data in this paper consisted of conversation during practical activity, and much of the decision making related directly to the activities underway, reminiscent of Beadle‐Brown's (2015) “person‐centred active support.” At times, both the PA and the person with intellectual disability were undertaking the same task, and at other times, they were doing something slightly different in the same physical space, for instance in the example from the craft workshop. Decisions and activity however were not always carried out in parallel. There were instances where people exerted their right to “executive” control (Boyle, 2008; Collopy, 1995) where a speaker asked the *other person *to enact their decision, as Wendy did in Extract 5, circumventing the difficulty of actually *doing *the actions that were necessary. We would thus add that:

- Supporting someone's autonomy does not always equate with encouraging them to perform a task. By following their request to do something, one can show respect for their executive autonomy.

There were also examples of more reflective talk about decisions, both past and future, in our data—where people with intellectual disabilities themselves sought out opportunities to discuss their lives. Where joint activities were in full flow, however, it was hard for both parties to stop and talk about future or present plans, to reflect on wise decisions or on the right to autonomy.

- People with intellectual disabilities may want to talk about past or future plans or decisions, and to reflect on their own reasoning. In a good relationship, this is often a key moment to stop and to listen supportively.

Maybe the central implication about relational autonomy arising from this research is that sequential positioning is vital. PAs need to listen and respond to the other person's turns in the conversation, and to ensure that body language, eye gaze and posture reflect that attempt to listen, points made strongly by the Misfits Theatre Company (2018). Routine strategies in any conversation, such as clarifying and checking out what the other person is saying (Kitzinger, 2014), might need to become more highly sensitized and adapted when interacting with a person whose speech is difficult to understand, such as Paul in our first two extracts. Social identities, such as being a competent decision maker, are constructed through talk with important others and thus:

- Body language and good listening are important, when the person being supported may want to talk about their future plans while doing something else.

Having autonomy to make decisions in a trusted relationship can mean that the person with intellectual disability simply trusts their PA to make the “right” decision for them. At that end of the spectrum of decision making, there are still learning opportunities which can be seized. However, the balance can be shifted, so that the decision is owned more by the person with intellectual disability. Some of the strategies highlighted in this paper, for instance, would be for the PA to admit that she does not always know best (see Extract 5) or to honour the epistemic right of the person they are supporting to know about their own life (see Extract 1).

The final point should go to the Misfits Theatre Company (2018), whose training film is all about having a “good match” between the person with intellectual disability and their PA. What is noticeable in all their examples is that, even when that good relationship exists, things can easily go wrong—with one awkward moment, an instruction or correction at the wrong point, or maybe a lapse of attention where the PA fails to notice what the person with intellectual disability is trying to say, possibly undermining the person with intellectual disability and casting them as incompetent. As the Misfits Theatre Company (2018) say in their film, successful support is not just in the hands of the PA; the person with intellectual disability themselves can take responsibility to ensure there is a “good match” with their PA. Not only did our data focus on the skills of PAs and support workers, but it underlined the initiations of people with intellectual disabilities who were active in seeking out chances to talk about, and make, their own decisions.

## ETHICAL APPROVAL

This research was carried out with full ethical approval from the Social Care Research Ethics Committee (SCREC) and with informed consent from all participants.

## CONFLICT OF INTEREST

There are no conflicts of interest to report.
